# COVID-19: Testing the Limits of Human Rights

**DOI:** 10.1017/err.2020.27

**Published:** 2020-04-07

**Authors:** Alessandra SPADARO

**Affiliations:** * PhD Candidate, Graduate Institute of International and Development Studies, Geneva, Switzerland; Teaching Assistant, Geneva Academy of International Humanitarian Law and Human Rights, Geneva, Switzerland; email: alessandra.spadaro@graduateinstitute.ch

## I. Introduction

“*Nous somme en guerre*”, proclaimed French President Emmanuel Macron, announcing a series of aggressive measures to contain the spread of the COVID-19 pandemic.^1^ References to “a war against the invisible enemy” and healthcare workers on the “frontline” have been made by US President Donald Trump,^2^ and other political leaders wanting to underscore the exceptional nature of the situation have also had recourse to war metaphors.^3^ In line with this rhetoric, it was even suggested that the outbreak should be designated as an armed attack for the purposes of Article 5 of the North Atlantic Treaty.^4^ Indeed, the war-like responses to the pandemic have been characterised by the taking of measures severely limiting the enjoyment of personal freedoms, to an extent that was unprecedented in democratic countries in times of peace. While taking different forms in various countries, the measures adopted broadly aim at enforcing social distancing among the population so as to minimise the human-to-human transmission of the new coronavirus that causes COVID-19. As a consequence, billions of people around the world have been put under some sort of lockdown.^5^ Concerns about the impact of such measures on human rights have been raised by the United Nations (UN) High Commissioner for Human Rights and other human rights experts.^6^ Such concerns are not unfounded, as measures restricting the enjoyment of human rights and the war rhetoric that accompanies them can open the way to the abuse of emergency regulations and the overreach of executive powers. Both the pandemic and the responses to it are putting to the test human rights, and not only in authoritarian countries.

In addressing this issue, this article starts by explaining why the taking of measures to contain the pandemic is warranted under human rights law. The article shows that, at the same time, some measures can have a detrimental effect on the enjoyment of a number of human rights. With a focus on the International Covenant on Civil and Political Rights (ICCPR) and on the European Convention on Human Rights (ECHR), the article then proceeds to analyse the conditions under which States may legitimately interfere with certain human rights through either limitations or derogations and highlights some areas of concern in this respect. It concludes that while the curtailment of certain freedoms might be temporarily necessary to deal with the COVID-19 outbreak, such curtailment should be carefully limited and constantly monitored so as to avoid abuses.

## II. The COVID-19 pandemic and containment measures in light of human rights law

The COVID-19 pandemic in itself threatens the enjoyment of human rights, most prominently the right to life and the right to health. It also highlights how human rights are interdependent while at the same time reflecting competing interests that are sometimes hard to reconcile.

The right to life is most evidently affected by the outbreak of COVID-19, which has already killed tens of thousands of individuals around the world. States have a due diligence obligation to protect individuals from deprivation of life caused by private persons.^7^ This due diligence obligation could be read as including protecting individuals from threats to life posed by others carrying an infectious and deadly disease, such as COVID-19. Indeed, the obligations of States to respect and ensure the right to life also encompass foreseeable threats and the taking of measures to address life-threatening diseases.^8^

The prevention and treatment of epidemics are also facets of the right to health, and so is access to healthcare.^9^ In this context, other relevant aspects of the right to health include access to safe and potable water and adequate sanitation, as well as access to adequate food, nutrition and housing.^10^ Questions regarding the respect of the right to a safe and healthy working environment, which is another declination of the right to health,^11^ notably arise with respect to healthcare workers involved in the care of COVID-19 patients.

While the fulfilment of the human right to the highest attainable standard of health is conditional on the resources that are available to a State,^12^ the COVID-19 pandemic is shedding light on the fact that the promotion of this right is fundamental for the fulfilment of other human rights, including civil and political rights. The link between the protection of the right to life and the protection of the right to health is the most obvious. In fact, the spread of an infectious disease that overwhelms the healthcare system threatens not only the lives of those who contract the disease and require medical care, but also the right to life and access to healthcare of individuals who continue to need treatment for other conditions.

If, on the one hand, the pandemic underlines the necessity of upholding the rights to life and health in order for the normal life of a democratic society to be preserved, on the other, it also shows the tension between these and other rights. This is because there are scarce resources to address and manage the pandemic, as well as competing individual and collective interests. In fact, public health measures consisting in the enforcement of social distancing, which are deemed effective in reducing the spread of certain influenza-like diseases, including COVID-19, clash with a number of individual rights. It is worth giving a few examples, based on some of the most commonly adopted measures, with no pretence of providing an exhaustive overview.^13^

One of the rights most clearly affected by the measures adopted by many States in response to the COVID-19 pandemic is freedom of movement. Countries in fact have restricted international travel, including by forbidding entry to non-nationals.^14^ Many countries have also restricted movement within their borders. For instance, Italy and France require individuals not to leave the house save in exceptional circumstances of necessity, such as for buying groceries or seeking medical care, and to justify their movements through a written declaration.^15^ So-called shelter-in-place orders have similarly been issued at the State level in the USA,^16^ while China went as far as imposing a total lockdown on millions of people.^17^

The enjoyment of the right to personal freedom is affected by the imposition of mandatory quarantine onto passengers coming from abroad and by the imposition of isolation onto people suspected or known to be positive for the new coronavirus.^18^ Prohibitions of public gatherings have an impact on the freedoms of assembly and association, while surveillance measures aimed at tracing contacts through the use of mobile data and other artificial intelligence tools pose a challenge to the full enjoyment of the right to a private life.^19^ The external dimension of the freedom to manifest one’s belief and religion is instead affected by the closure of places of worship. The closure of businesses and workplaces also has consequences on the enjoyment of the right to work, especially by workers in the informal economy^20^ and more generally by those who cannot work from home, while the closure of schools and universities affects the right to education.

## III. Limitations and derogations

The question naturally arises of whether the measures briefly outlined above are legitimate. Some human rights (such as the freedom from torture and slavery) are absolute and allow for no limitations, balancing with other rights or derogations. However, most human rights are not absolute and can be restricted, albeit within certain limits. Human rights treaties specifically envision two tools that can and are being used by States to take measures to manage the COVID-19 pandemic that interfere with some human rights: limitations and derogations.

Limitations allow precisely for the balancing of individual and collective interests and are built into several provisions of the ICCPR and of the ECHR and its Protocols. Limitations to non-absolute rights are allowed when they are prescribed by law, pursuant to a legitimate aim and when such limitations are necessary in a democratic society and proportionate to the identified legitimate aim, meaning that no other less restrictive alternative is available.^21^

While worded in slightly different ways, both the ECHR and the ICCPR identify certain legitimate aims as grounds for limiting a series of rights, such as the right to respect for private and family life (Article 8 ECHR), freedom to manifest one’s religion or belief (Article 9 ECHR and Article 18 ICCPR), freedom of expression (Article 10 ECHR and Article 19 ICCPR), freedom of assembly and association (Article 11 ECHR and Articles 21 and 22 ICCPR) and freedom of movement (Article 2 ECHR Protocol no. 4 and Article 12 ICCPR). Public health is one of the identified legitimate aims that “may be invoked as a ground for limiting certain rights in order to allow a State to take measures dealing with a serious threat to the health of the population or individual members of the population”.^22^

In times of public emergency threatening the life of a nation, the possibility for States to derogate from some of their obligations under human rights law is also envisioned. Derogations effectively consist in the temporary suspension of certain human rights and are thus allowed only to the extent that they are strictly required by the exigencies of the situation and are not inconsistent with the State’s other obligations under international law, including the principle of non-discrimination. Derogations must also follow the notification procedures outlined in Article 4 ICCPR and Article 15 ECHR, respectively, which require that the state of emergency be publicly proclaimed and appropriately communicated.

With regards to the use of limitations and derogations, the Human Rights Committee explained that:

If States purport to invoke the right to derogate from the Covenant during, for instance, a natural catastrophe, a mass demonstration including instances of violence, or a major industrial accident, they must be able to justify not only that such a situation constitutes a threat to the life of the nation, but also that all their measures derogating from the Covenant are strictly required by the exigencies of the situation. In the opinion of the Committee, the possibility of restricting certain Covenant rights under the terms of, for instance, freedom of movement (art. 12) or freedom of assembly (art. 21) is generally sufficient during such situations and no derogation from the provisions in question would be justified by the exigencies of the situation.^23^

These considerations would also seem applicable in the case of a pandemic, which was not explicitly considered by the Committee. Similarly, in order for a derogation from the ECHR to be possible, there would need to exist an actual or imminent public emergency, involving the whole nation, threatening the organised life of the community and be exceptional, “in that the normal measures or restrictions, permitted by the Convention for the maintenance of public safety, health and order, are plainly inadequate”.^24^

Limitations and derogations can be seen as a continuum. In this vein, States should have recourse to the latter only as a last resort, when limitations have proven to be manifestly insufficient to respond to a public emergency.^25^ Derogations might become necessary the longer the restrictions last, since limitations “of a long duration are particularly likely to be disproportionate to the legitimate aim pursued”.^26^

Speaking about the response to natural disasters, one scholar suggested that States might use derogations, even when limitations would suffice, when in doubt as to whether the measures taken might be in violation of their human rights obligations.^27^ One might go even further and argue that, if mere limitations on public health grounds were sufficient but derogations were nevertheless adopted, this could suggest an attempt to abuse emergency powers by suspending certain human rights.

On the other hand, it should be stressed that derogations are in the prerogative of States in times of public emergencies threatening the life of a nation, and a pandemic could certainly be considered as such. This assessment is further bolstered by the likening of the pandemic to a war, which is the paradigmatic example of a public emergency threatening the life of a nation. Nor should it be assumed that a State derogating from its obligations automatically violates the human rights of the individuals under its jurisdiction, while States simply limiting human rights on public health grounds do not. Indeed, one might argue that derogations are the most appropriate tool to deal with situations of emergency. Albeit in a different context, the UN Special Rapporteur on counterterrorism and human rights has underscored that derogations are “necessary for transparency and accountability when States exercise emergency powers”,^28^ and that failure to derogate often results in de facto emergencies subtracted to domestic and international legal oversight of emergency powers.^29^ At the time of writing, and in spite of the widespread use of the war rhetoric, only a handful of States have notified their intention to derogate from some of their obligations under the ICCPR and the ECHR due to the COVID-19 emergency. In at least certain cases, the absence of a derogation might signify that the government believes that the situation – albeit extraordinary – can be dealt with by simply limiting human rights on public health grounds. In others, it might be indicative of an attempt to subtract the measures adopted from the scrutiny of the international community.

## IV. Limiting and monitoring interferences with human rights in the context of the COVID-19 pandemic

Regardless of whether they take the form of limitations or derogations, interferences with fundamental human rights should be viewed with caution, if not suspicion, and they should be strictly limited, both materially and temporally, to what is required to address the COVID-19 pandemic. Most definitely, they should not be used to promote power grabs, quash dissent or persecute minorities.

In order to prevent the curtailment of human rights from becoming the new normal, States should strive to adopt a long-term strategy for the management of the pandemic that does not rely on the continued restriction or suspension of fundamental freedoms. They should also be wary of the long-lasting and sweeping effects of certain measures. For instance, the use of mobile data and artificial intelligence for contact tracing raises concerns regarding the storage and use of the collected data both during and after the pandemic, as these tools could be abused for political purposes in order to implement mass surveillance of the population, going well beyond the needs of tracking the spread of the pandemic.

The publicity of the measures is also crucial, whether they are adopted through limitations or derogations. The latter in fact require an official proclamation, while limitations must be provided by law, which must be clear and accessible to everyone.^30^ This requirement is essential to prevent abusive interpretations and applications of the law and to ensure that individuals are precisely informed of what they are expected to do. In this respect, it is worth noting that two rights that should neither be limited nor suspended as part of the measures to tackle the COVID-19 pandemic are the right to information and the right to freedom of expression. Indeed, China’s failure to uphold these rights seems to have delayed both the Chinese and the global response to the pandemic.^31^ The World Health Organization (WHO) has underscored the importance of the public’s right to information in order to successfully manage the pandemic, because it allows the sensitisation of the population as to the health risks posed by COVID-19 and as to the strategies to mitigate them.^32^ In the context of this pandemic, the right to information should also be read as encompassing the communication of truthful and complete data regarding the number of cases and deaths due to COVID-19. Crucially, the continued upholding of the right to information and the right to freedom of expression also allows for the constant monitoring of the legitimacy, necessity and proportionality of the containment measures taken by the government in relation to their impact on human rights. Whatever tool is chosen by governments to temporarily interfere with the enjoyment of some fundamental rights in the wake of the COVID-19 outbreak, the public and democratic oversight of the measures taken, at the national and international level, is essential to ensuring that the use of emergency powers is not normalised and that the restricted rights can expand again to their original form as soon as possible. In this sense, the absence of a sunset clause in a recent law passed in Hungary, which gives Prime Minister Viktor Orbán the power to rule indefinitely by decree without the oversight of Parliament, is particularly worrying.^33^

The role of national and international courts and of the concerned treaty bodies is also decisive in ensuring the respect of human rights during the COVID-19 pandemic. In terms of judicial oversight, the effects of either limitations or derogations are similar but not identical. If human rights are limited, substantive claims brought by individuals complaining about the restrictions adopted can be adjudicated in terms of their legality, necessity and proportionality with regards to the identified legitimate aim. In the case of derogations, a national court or the concerned treaty body would first enquire whether the conditions for a derogation were met, and, if not, find a violation of the human rights in question. If the derogation seems to be justified, the national court or treaty body would examine whether the measures complied with other relevant rules of international law and were “strictly required by the exigencies of the situation”. Were this not the case, the State would have violated the human rights affected by the derogation.^34^ In either instance, courts normally show a certain amount of deference to the assessment made by the State regarding the necessity of interfering with human rights. Should cases arise in the future to challenge the measures taken with respect to the COVID-19 pandemic, courts and treaty bodies might need to have recourse to public health expertise to assess whether the measures taken were in fact necessary. For instance, they might take into account that the WHO advised States to adopt an integrated approach to tackling the COVID-19 pandemic, which comprises not only the taking of public health measures enforcing social distancing, which have been found to be effective,^35^ but also the thorough diagnosis, monitoring and reporting of cases through the massive administration of tests, as well as the tracing of contacts to identify the chain of transmission and the isolation of ill individuals in separate structures.^36^ Indeed, the Republic of Korea seems to have adopted mainly the latter type of measures in lieu of strict social distancing measures in the form of lockdowns and has been extremely successful in curbing the incidence of deaths due to COVID-19.^37^ Therefore, if it appeared that a State, based on available resources, could have adopted an integrated approach allowing for the management of the pandemic to be equally – if not more – effective through less stringent restrictions on the enjoyment of fundamental human rights, the assessment of the necessity of limitations or derogations would have to be adjusted accordingly.

## V. Conclusion

This article has shown that States have an obligation, under human rights law, to tackle the COVID-19 pandemic. However, some of the containment measures adopted by different countries also imply severe interferences with a number of human rights, including – but not limited to – freedom of movement, the right to personal liberty, freedom of assembly and association, the right to a private life, the right to manifest one’s belief or religion, the right to work and the right to education. Human rights law allows for the limitations of certain non-absolute rights for the protection of public health. This article has shown that States can in fact use limitations to address the COVID-19 pandemic. If States deem limitations to be insufficient due to the exceptional character of the situation, they can also resort to derogations. However, in spite of the widespread likening of the pandemic to a war, few States so far have derogated from their obligations under the relevant human rights treaties. Provided that the conditions and requirements for having recourse to either limitations or derogations are respected, States can legitimately have recourse to either tool. However, whatever the reasons for which States may choose either limitations or derogations, it is of paramount importance that the measures taken are limited to what is strictly necessary to manage the pandemic and do not lead to the permanent curtailment of human rights. Indeed, constant scrutiny should be applied by courts and legislative bodies, the international community and civil society to all governmental initiatives regarding this global health crisis. The COVID-19 pandemic might well mark the end of the world as we know it, but we would not want to wake up in a new world where human rights have lost all significance.

## Footnotes

1 “Addresse aux Français du Président de la République Emmanuel Macron”, 16 March 2020 <https://www.elysee.fr/emmanuel-macron/2020/03/16/adresse-aux-francais-covid19>.

2 “Remarks by President Trump in a Meeting with Supply Chain Distributors on COVID-19”, 29 March 2020 <https://www.whitehouse.gov/briefings-statements/remarks-president-trump-meeting-supply-chain-distributors-covid-19>.

3 Eg “PM Says Greece At War with ‘Invisible Enemy’ Coronavirus”, *Reuters*, 17 March 2020 <https://www.reuters.com/article/us-health-coronavirus-greece-pm/pm-says-greece-at-war-with-invisible-enemy-coronavirus-idUSKBN2142T5>; G Erebara, “Albania Announces New ‘War’ Measures against Coronavirus”, *Balkan Insight*, 12 March 2020 <https://balkaninsight.com/2020/03/12/albania-close-factories-impose-curfew-in-war-against-new-coronavirus>.

4 F Kempe, ‘Why Trump Should Trigger NATO’s Article 5 vs. COVID-19”, *Atlantic Council*, 14 March 2020 <https://www.atlanticcouncil.org/content-series/inflection-points/why-trump-should-trigger-natos-article-5-vs-covid-19>.

5 H Davidson, “Around 20% of Global Population under Coronavirus Lockdown”, *The Guardian*, 24 March 2020 <https://www.theguardian.com/world/2020/mar/24/nearly-20-of-global-population-under-coronavirus-lockdown>.

6 “Coronavirus: Human Rights Need to Be Front and Centre in Response, Says Bachelet”, *OHCHR*, 6 March 2020 <https://www.ohchr.org/EN/NewsEvents/Pages/DisplayNews.aspx?NewsID=25668&LangID=E>; “COVID-19: States Should Not Abuse Emergency Measures to Suppress Human Rights – UN Experts”, *OHCHR*, 16 March 2020 <https://www.ohchr.org/EN/NewsEvents/Pages/DisplayNews.aspx?NewsID=25722>.

7 UN Human Rights Committee, “General Comment no. 36 (2018) on Article 6 of the International Covenant on Civil and Political Rights, on the Right to Life”, 30 October 2018, UN Doc CCPR/C/GC/36, paras 7, 21.

8 ibid, paras 7, 21, 26.

9 UN Committee on Economic, Social and Cultural Rights, “CESCR General Comment No. 14: The Right to the Highest Attainable Standard of Health (Art. 12)”, 11 August 2000, UN Doc E/C.12/2000/4, paras 12, 16.

10 ibid, para 11.

11 ibid, paras 4, 15.

12 ibid, para 9; UN Committee on Economic, Social and Cultural Rights, “CESCR General Comment No. 3: The Nature of States Parties’ Obligations (Art. 2, Para 1, of the Covenant)”, 14 December 1990, UN Doc E/1991/23, para 11.

13 For more details about the various governmental responses to the COVID-19 pandemic affecting human rights, see “COVID-19 Civic Freedom Tracker” <https://www.icnl.org/covid19tracker>.

14 For an overview of travel restrictions in the European Union, see “Coronavirus Response: Transport Measures” <https://ec.europa.eu/transport/coronavirus-response_en>.

15 See eg Décret no 2020-293 du 23 mars 2020 prescrivant les mesures générales nécessaires pour faire face à l’épidémie de covid-19 dans le cadre de l’état d’urgence sanitaire, Art. 3; Directive no. 14606 of 8 March 2020 of the Italian Ministry of Interior.

16 See eg Executive Order N-33-20, Executive Department, State of California, 19 March 2020.

17 E Graham-Harrison and L Kuo, “China’s Coronavirus Lockdown Strategy: Brutal but Effective”, *The Guardian*, 19 March 2020 <https://www.theguardian.com/world/2020/mar/19/chinas-coronavirus-lockdown-strategy-brutal-but-effective>.

18 See eg The Health Protection (Coronavirus) Regulations 2020 No. 129, ss 4–9, specifically referring to “isolation and detention”. It should be noted that “the lawful detention of persons for the prevention of the spreading of infectious diseases” is one of the six lawful grounds of detention exhaustively identified by Art 5 ECHR. The lawfulness of such detention depends on “whether the spreading of the infectious disease is dangerous to public health or safety, and whether detention of the person infected is the last resort in order to prevent the spreading of the disease, because less severe measures have been considered and found to be insufficient to safeguard the public interest” (*Enhorn v Sweden* App no. 56529/00 (ECtHR Second Section, 25 January 2005), para 44).

19 See eg B Trew, “Coronavirus: Controversial Israeli Spyware Firm NSO Builds Software Tracking Mobile Data to Map Covid-19”, *The Independent*, 18 March 2020 <https://www.independent.co.uk/news/world/middle-east/coronavirus-israel-cases-tracking-mobile-phone-nso-spyware-covid-19-a9410011.html>; S Yuan, “How China Is Using AI and Big Data to Fight the Coronavirus’, *Al Jazeera*, 1 March 2020 <https://www.aljazeera.com/news/2020/03/china-ai-big-data-combat-coronavirus-outbreak-200301063901951.html>.

20 For instance, on the impact of the lockdown on informal economy workers in India, see “India: COVID-19 Lockdown Puts Poor at Risk”, *Human Rights Watch*, 27 March 2020 <https://www.hrw.org/news/2020/03/27/india-covid-19-lockdown-puts-poor-risk>.

21 General Comment no. 14, supra, note 9, paras 28, 29; UN Human Rights Committee, “General Comment No. 31: The Nature of the General Legal Obligation Imposed on States Parties to the Covenant”, 26 May 2004, UN Doc CCPR/C/21/Rev.1/Add. 13, para 6.

22 UN Economic and Social Council, “The Siracusa Principles on the Limitation and Derogation Provisions in the International Covenant on Civil and Political Rights”, 28 September 1984, UN Doc E/CN.4/1985/4, para 22.

23 UN Human Rights Committee, “General Comment no. 29: Article 4: Derogations during a State of Emergency”, 31 August 2001, UN Doc CCPR/C/21/Rev.1/Add.11, at para 5. See also Siracusa Principles, supra, note 22, para 39.

24 *The Greek Case*, Apps no. 3321/67, 3322/67, 3323/67, 3344/67 (European Commission of Human Rights, Report of the Sub-Commission, Volume I, Part 1, 1969), para 113. The European Court of Human Rights seems willing to accept interferences with the rights protected by the Convention in case of “the existence of an exceptional crisis without precedent”, even in the absence of derogations: see *Koufaki and Adedy v Greece*, Apps no. 57655/12 and 57657/12 (ECHR First Section, 7 May 2013), para 37.

25 G Giacca, Economic, Social, and Cultural Rights in Armed Conflict (Oxford, Oxford University Press 2014) pp71, 107.

26 *Kuimov v Russia* App no. 32147/04 (ECHR First Section, 8 January 2009), para 96.

27 E Sommario, “Limitation and Derogation Provisions in International Human Rights Law Treaties and Their Use in Disaster Settings” in F Zorzi Giustiniani, E Sommario, F Casolari and G Bartolini (eds), Routledge Handbook of Human Rights and Disasters (Abingdon, Routledge 2018) p 113.

28 “Report of the Special Rapporteur on the Promotion and Protection of Human Rights and Fundamental Freedoms while Countering Terrorism on the Human Rights Challenge of States of Emergency in the Context of Countering Terrorism”, 1 March 2018, UN Doc. A/HRC/37/52, para 22.

29 ibid, para 27.

30 Siracusa Principles, supra, note 22, para 17.

31 L Yuan, “China Silences Critics over Deadly Virus Outbreak”, *New York Times*, 22 January 2020 <https://www.nytimes.com/2020/01/22/health/virus-corona.html>.

32 WHO, “Responding to Community Spread of COVID-19: Interim Guidance”, 7 March 2020 <https://apps.who.int/iris/handle/10665/331421>.

33 I Tharoor, “Coronavirus Kills Its First Democracy”, *The Washington Post*, 31 March 2020, <https://www.washingtonpost.com/world/2020/03/31/coronavirus-kills-its-first-democracy>.

34 L Doswald-Beck, Human Rights in Times of Conflict and Terrorism (Oxford, Oxford University Press 2011) p 89.

35 See generally *Report of the WHO–China Joint Mission on Coronavirus Disease 2019 (COVID-19)*, February 2020, pp 16–24 <https://www.who.int/docs/default-source/coronaviruse/who-china-joint-mission-on-covid-19-final-report.pdf>.

36 See WHO, supra, note 32.

37 A Dudden and A Marks, “South Korea Took Rapid, Intrusive Measures against Covid-19 and They Worked”, *The Guardian*, 20 March 2020, <https://www.theguardian.com/commentisfree/2020/mar/20/south-korea-rapid-intrusive-measures-covid-19>.

